# Mechanisms of enhanced antiglioma efficacy of polysorbate 80‐modified paclitaxel‐loaded PLGA nanoparticles by focused ultrasound

**DOI:** 10.1111/jcmm.13695

**Published:** 2018-06-29

**Authors:** Yingjia Li, Manxiang Wu, Nisi Zhang, Caiyun Tang, Peng Jiang, Xin Liu, Fei Yan, Hairong Zheng

**Affiliations:** ^1^ Department of Medicine Ultrasonics Nanfang Hospital Southern Medical University Guangzhou China; ^2^ Paul C. Lauterbur Research Center for Biomedical Imaging Institute of biomedical and Health Engineering Shenzhen Institutes of Advanced Technology Chinese Academy of Sciences Shenzhen China; ^3^ Biomedical Engineering Department College of Engineering Peking University Beijing China; ^4^ Pharmaceutical Analysis Department College of Pharmacy Jiamusi University Jiamusi China; ^5^ Shenzhen Key Laboratory of Nanobiomechanics Shenzhen Institutes of Advanced Technology Chinese Academy of Sciences Shenzhen China

**Keywords:** blood‐brain barrier, drug delivery, focused ultrasound, glioma, polysorbate 80

## Abstract

The presence of blood‐brain barrier (BBB) greatly limits the availability of drugs and their efficacy against glioma. Focused ultrasound (FUS) can induce transient and local BBB opening for enhanced drug delivery. Here, we developed polysorbate 80‐modified paclitaxel‐loaded PLGA nanoparticles (PS‐80‐PTX‐NPs, PPNP) and examined the enhanced local delivery into the brain for glioma treatment by combining with FUS. Our result showed PPNP had good stability, fast drug release rate and significant toxicity to glioma cells. Combined with FUS, PPNP showed a stronger BBB permeation efficiency both in the in vitro and in vivo BBB models. Mechanism studies revealed the disrupted tight junction, reduced P‐glycoprotein expression and ApoE‐dependent PS‐80 permeation collectively contribute to the enhanced drug delivery, resulting in significantly stronger antitumour efficacy and longer survival time in the tumour‐bearing mice. Our study provided a new strategy to efficiently and locally deliver drugs into the brain to treat glioma.

## 1 INTRODUCTION

The past decades have seen great progress in the drug treatment for most of tumours, but really limited for glioma which still remains high rates of recurrence and mortality.^1^, ^2^, ^3^, ^4^, ^5^, ^6^, ^7^ The blood‐brain barrier (BBB), a highly selective semipermeable membrane barrier that separates the circulating blood from the brain and extracellular fluid in the central nervous system, is considered to be one of the most important obstacles to drug therapy for glioma. Under normal physiological conditions, BBB allows the passage of materials crucial to neural function such as water, some gases, glucose and amino acids by passive diffusion or selective transport. Meanwhile, it also prevents the entry of potential neurotoxins by the way of an active transport mechanism mediated by P‐glycoprotein. In some cases, drugs have to be administered directly into the cerebrospinal fluid (CSF), where it can enter the brain by crossing the blood‐cerebrospinal fluid barrier.^8^, ^9^ However, not all drugs that are delivered directly to the CSF can effectively penetrate the CSF barrier and enter the brain, severely restricting the availability for most anticancer agents.^10^, ^11^ Therefore, it is desirable to develop new techniques to selectively deliver a chemotherapeutic agent into brain tumour for glioma therapy.^12^

To date, many strategies have been applied to deliver drug across the BBB, including the intra‐arterial injection of hyperosmotic fluids (such as mannitol) to induce osmotic blood‐brain barrier disruption, the design of drug delivery systems targeted to transport receptors highly expressed at the BBB (such as transferrin receptor) or using a naturally occurring compound as adjuvant (such as borneol) to improve drug delivery to the brain.^13^, ^14^, ^15^, ^16^, ^17^ Recently, polysorbate 80 (PS‐80), known as a surfactant, was identified to be able to enhance the BBB transport by modifying nanoparticles.^18^, ^19^, ^20^ Researchers demonstrated the possible mechanism is that PS‐80 can selectively adsorb apolipoprotein E (ApoE) on the brain capillary endothelium and induce low‐density lipoprotein receptor‐related protein (LRP) mediated transcytosis across the BBB.^21^, ^22^, ^23^ However, drug delivery system mediated with PS‐80 often produces whole brain but not local drug delivery, resulting in undesired drug accumulation at health sites and inadequate drug concentrations in the tumour region. In addition, PS‐80‐modified nanoparticles would be ineffective for these glioma patients who do not express or only express low level of ApoE proteins.

Recently, focused ultrasound (FUS) combined with microbubbles (MBs) provides a promising approach to achieve local delivery into the brain.^12^, ^24^ Taking advantage of MB cavitation induced by FUS, transient and reversible BBB opening can be induced, without obvious damage to the cellular ultrastructure.^25^, ^26^ Also, US transducer is portable and US beams can be easily focused on the brain tumour site, giving the approach great advantages in the local drug delivery into brain. So far, FUS combined with MBs have successfully delivered drugs, genes, proteins and cells for brain tumour treatment.^13^, ^27^, ^28^, ^29^, ^30^, ^31^ In this study, paclitaxel (PTX), a chemotherapeutic agent that can hardly cross the BBB, was chosen as a model drug. Herein, we fabricated PS‐80‐modified PTX‐loaded PLGA nanoparticles (PS‐80‐PTX‐PLGA, PPNP) and locally delivered it into brain for the enhanced antitumour efficacy through combining with FUS irradiation. The preparation, characterization, in vitro and in vivo drug delivery efficiency across BBB, and antitumour effect of PPNP were investigated. Importantly, the mechanisms of penetration through BBB were also explored in the study.

## 2 MATERIALS AND METHODS

### 2.1 Materials

Poly (lactic‐co‐glycolic acid) (PLGA) (50:50, MW 24 000‐38 000), poly (vinyl alcohol) (PVA) (50:50, MW 30 000‐70 000), polysorbate 80 (Tween‐80, PS‐80), PTX, Evans blue (EB), IR‐780 and Coumarin‐6 dyes were obtained from Sigma‐Aldrich (St. Louis, MO, USA). Cell Counting Kit‐8 (CCK‐8) was purchased from Dojindo Laboratories (Tokyo, Japan). The in situ Cell Death Detection Kit and DAPI were obtained from Roche (Mannheim, Germany). Anti‐ApoE antibody (ab1906) and rabbit antimouse IgG H&L (HRP) (ab6728) were purchased from Abcam (Cambridge, USA). Human glioblastoma U87‐Luc and murine brain vein endothelial bEnd.3 cell lines were purchased from the American Type Culture Collection. Female BALB/c nude mice, weighing about 20 g (6‐8 weeks old), were obtained from Guangdong Medical Experimental Animal Center (Guangzhou, China). All other reagents were of analytical grade.

### 2.2 Preparation of PPNP

PTX‐loaded PLGA nanoparticles (PTX‐PLGA‐NPs, PNP) were prepared following the emulsion solvent evaporation method with slight modifications. Briefly, PTX and PLGA, dissolved in 1 mL of dichloromethane, were added dropwise to 5% w/v aqueous PVA solution (5 mL), followed by sonication for 5 minutes (30% amplitude, 50 ms on, 10 ms off) to generate water/oil emulsion. This emulsion was stirred at 700 rpm for 6‐8 hours until complete removal of organic solvent, followed by centrifugation at 9 500 g for 10 minutes at 4°C. The pellet obtained was resuspended in water and washed twice. The nanoparticles were freeze‐dried and stored at 4°C under anhydrous conditions. To obtain the PS‐80‐coated PTX‐loaded PLGA, PNP formulation was treated as the procedure described by Wilson et al.^32^ In brief, PNP were resuspended in water and PS‐80 was added to 1% PS‐80 final concentration; then, the mixture was incubated for 30 minutes. The resulting PPNP were collected by centrifugation and finally lyophilized. For visualization, PS‐80‐coated IR‐780‐loaded PLGA NPs (PS‐80‐IR‐780‐NPs, PINP) and PS‐80‐coated Coumarin‐6‐loaded PLGA NPs (PS‐80‐Cou‐6‐NPs, PCNP) were also fabricated using IR‐780 or Coumarin‐6 instead of PTX drugs.

### 2.3 In vitro PTX release

In vitro PTX release from PPNP was studied in 0.02 mol/L phosphate‐buffered saline (PBS, pH 7.4) with 0.5% PS‐80. In a dialysis tubing, approximately PPNP equivalent to 1 mg PTX were suspended in 2 mL distilled water. Then, the dialysis tubing was placed into 250 mL medium at 37°C, followed by continuous shaking at 100 rpm and 37°C in a constant temperature shaking bath. Samples (1 mL) were withdrawn at predetermined time intervals for HPLC analysis and replaced with an equal volume of fresh PBS. The release rate (RR) was calculated according to the equation: RR% = (*W*_i_/*W*_total_) × 100%, where *W*_i_ is the measured amount of PTX at the indicated time point and *W*_total_ is the total PTX amount in the same volume of NPs suspensions.

### 2.4 Cell viability

In order to investigate toxicity to tumour cells, we treated U87 cells with PPNP or free PTX at 0, 3, 6, 9, 12 and 24 μg/mL of PTX concentrations. After 3 days, the media were replaced with 110 μL of fresh solution (100 μL medium plus 10 μL of CCK‐8 solution). After 45 min of incubation, the CCK‐8 assay was performed. Cell viability in each group was expressed as a percentage relative to that of the untreated control.

### 2.5 In vitro BBB model

bEnd.3 cells were seeded into transwell to establish the in vitro blood‐brain barrier model. Briefly, transwell permeable supports with 0.4‐μm porous membranes (Corning Inc., NY, USA) were used. bEnd.3 cells were seeded into the support at 5 × 10^4^ cells per support and incubated for 48 hours. The culture media was replaced every 3 days. After 1 week, sodium fluorescein was used as probe to measure the paracellular or junctional permeability. To calculate endothelial permeability coefficient (Pe), flux of the sodium fluorescein across monolayers and cell‐free inserts is measured^33^, ^34^: Clearance (μL) = [Concentration_abluminal_] × [Volume_abluminal_] × [Concentration_luminal_]^−1^. The average volume cleared is plotted versus time, and permeability × surface area product value for endothelial monolayer (PSe) is calculated by the following formula: PS_endothelial_^−1^ = PS_time‐plotted_^−1^ − PS_insert_^−1^, PS_time‐plotted_ is the slope of the monolayer group. PS_insert_ is the slope of cell‐free group. PSe divided by the surface area generates the endothelial permeability coefficient [Pe (cm/s)] for sodium fluorescein.

### 2.6 Ultrasound equipment

The sonications were performed using a single‐element focused ultrasound (FUS) transducer (centre frequency: 1 MHz, transducer diameter: 43.1 mm, curvature radius: 44.45 mm). In the in vitro experiment, the transducer was fixed in a water tank to make sure that the focus of the ultrasound beam was positioned to the desired region. In the in vivo experiment, the transducer was mounted on a removable cap filled with deionized and degassed water whose tip was covered by a thin and transparent sealing film and the centre of the focal spot was about 4.0 mm away from the cap tip. The peak negative pressure amplitude was measured in a water tank with a 0.8 mm diameter needle hydrophone (PT; Acoustical Institute of the Chinese Academy of Sciences, Beijing, China).

### 2.7 In vitro BBB penetration

A given amount of CNP or PCNP (equal Coumarin‐6) were added into transwell insert which had been developed the in vitro BBB model. To ensure a close contact between MBs and cells, the transwells were sealed with sealing film and inverted. The transwell was exposed under 1.26 W/cm^2^ for 60 seconds. The microbubbles that have good stability were obtained according to our previous study.^35^, ^36^ Fluorescence intensity of abluminal medium was measured at 1, 2, 4, 8 and 12 hours after FUS exposure. In order to further determine the mechanism of PS‐80 in the BBB penetration, nanoparticles with or without PS‐80 were used to evaluate the BBB permeability. First, bEnd.3 cells were seeded in 12‐well plate at a density of 1 × 10^5^ cells per well in medium and incubated for 24 hours. Then, the cells were treated with PCNP or CNP (equal Coumarin‐6) for 0.5 or 2 hours. The fluorescence images were observed by fluorescence microscopy. The incubated cells were washed 3 times with cold PBS, detached by trypsinization and then suspended in PBS. The suspended cells were directly detected by flow cytometer.

### 2.8 In vivo BBB opening

Before sonication, the mice were anaesthetized with 1.5% isoflurane (RWD Life Science, Co., Ltd., Shenzhen, China) and placed on a heating pad with a constant temperature of 37°C to maintain their body temperature. A bolus (1.25 × 10^8^ bubbles/kg) MBs was administered intravenously at the beginning of the sonication. Evans blue (100 mg/kg) extravasation and HE staining were used to evaluate the feasibility of FUS‐induced BBB opening. After extensive preliminary experiments, we chose 2.9 W/cm^2^ and 60 seconds as the final applied parameters and the peak negative pressure is 0.3 MPa, which was similar to the previous report.^37^ To visualize the vivo BBB opening, healthy Balb/c nude mice were treated with saline, INP, PINP, INP + FUS and PINP + FUS, respectively. At predetermined time‐points, these mice were imaged using the Xenogen IVIS spectrum imager (Caliper Life Sciences, Inc., Hopkinton, MA) at 745 nm excitation and 820 nm emission wavelengths.

### 2.9 Immunofluorescence assays

Immunofluorescence staining was performed to detect expression and localization of ZO‐1 and P‐glycoprotein (P‐gp). The cells or brain slices were fixed with 4% paraformaldehyde solution and permeabilized with 0.1% Triton X‐100, followed by blocking with PBS containing 1% BSA for 1 hour at room temperature. After that, the cells or slices were incubated with goat polyclonal anti‐ZO‐1 antibody (1:50 dilution; C‐19, Santa Cruz) or mouse monoclonal P‐glycoprotein antibody (1:50 dilution; C219, Thermofisher) at 4°C overnight, respectively. After a series of washes in PBS, the cells or slices were stained with donkey anti‐goat IgG H&L secondary antibody (FITC, 1:200 dilution; ab6881, Abcam) or goat antimouse IgG H&L preadsorbed secondary antibody (Alexa Fluor^®^405, 1:200 dilution; ab175661, Abcam) for 1 hour at room temperature, respectively. The cells or slides were washed 3 times with PBS, incubated with DAPI for 15 minutes and then examined under a fluorescence microscope.

### 2.10 Animal models

Female BALB/c nude mice (6‐8 weeks) were obtained from Guangdong Medical Experimental Animal Center (Guangzhou, China). Animals received care in accordance with the Guidance Suggestions for the Care and Use of Laboratory Animals. The procedures were approved by Shenzhen Institutes of Advanced Technology, Chinese Academy of Sciences Animal Care and Use Committee. For the intracranial implant of U87‐Luc tumours, cells were suspended in ice‐cold PBS at 1 × 10^8^ cells/mL. Mice were anaesthetized with isoflurane and attached to the base of a stereotaxic frame with ear bars. The skull was exposed through a 1‐cm midline incision, and a burr hole was made 2 mm to the right of the bregma and 1.3 mm posterior to the coronal suture using a syringe with a 1‐mm tip. Using a microsyringe attached to the stereotaxic frame, 5 μL of U87‐Luc cells (1 × 10^5^ cells) was injected over 5 min at a depth of 3 mm. After the injection, the syringe was kept in place for 1 minute prior to withdrawal and the incision was closed with Vetbond skin glue. The animals were monitored for tumour growth using an in vivo imaging system (IVIS Lumina II, Caliper, USA).

### 2.11 In vivo antitumour efficacy

Ten days after intracranial inoculation of U87‐Luc cells, the in vivo bioluminescence imaging was used to determine whether the tumour‐bearing models were established successfully. Sixty nude mice were randomly divided into 5 groups (Saline, PNP, PPNP, PNP+FUS and PPNP+FUS, n = 12). Microbubbles and nanoparticles were co‐injected. The amount of microbubbles and nanoparticles was 1.25 × 10^8^ bubbles/kg and 3 mg/kg of PTX concentrations. Treatment was performed once every 3 days via tail vein injection of the nanoparticles with or without FUS irradiation. The in vivo bioluminescence imaging was used to monitor the growth of tumour with an IVIS Spectrum system (Caliper Life Sciences, Inc., Hopkington, MA).

### 2.12 Histological analysis

At the end of the treatment, the mice were sacrificed and tumours were excised for histological analysis. Organ tissues including liver, heart, lung, spleen and kidney were collected for haematoxylin and eosin (H&E) staining. All tissues were prepared and sectioned to standard procedures. For the H&E staining, tissues were then embedded in paraffin wax, cut into 5‐μm‐thick sections and stained with haematoxylin and eosin dyes. Microscopic images of the tissues were acquired using an optical microscope. For immunofluorescence staining, tissues were embedded in OCT, cut into 8‐μm‐thick sections. Then slides were stained with anti‐ki67 antibodies to assess tumour proliferation. Tumour apoptosis was also assessed by TUNEL assay according to the product instruction.

### 2.13 Statistical analysis

Statistical analysis was carried out with SPSS version 23.0 (SPSS Inc., Chicago, IL). All values were expressed as means ± SD. Data were analysed by ANOVA, and then, differences among the means were analysed using the Tukey‐Kramer multiple comparison test. Differences were considered significant at *P < .05 and very significant at **P < .001.

## 3 RESULTS

### 3.1 Fabrication and characterization of PPNP

**Figure 1:**
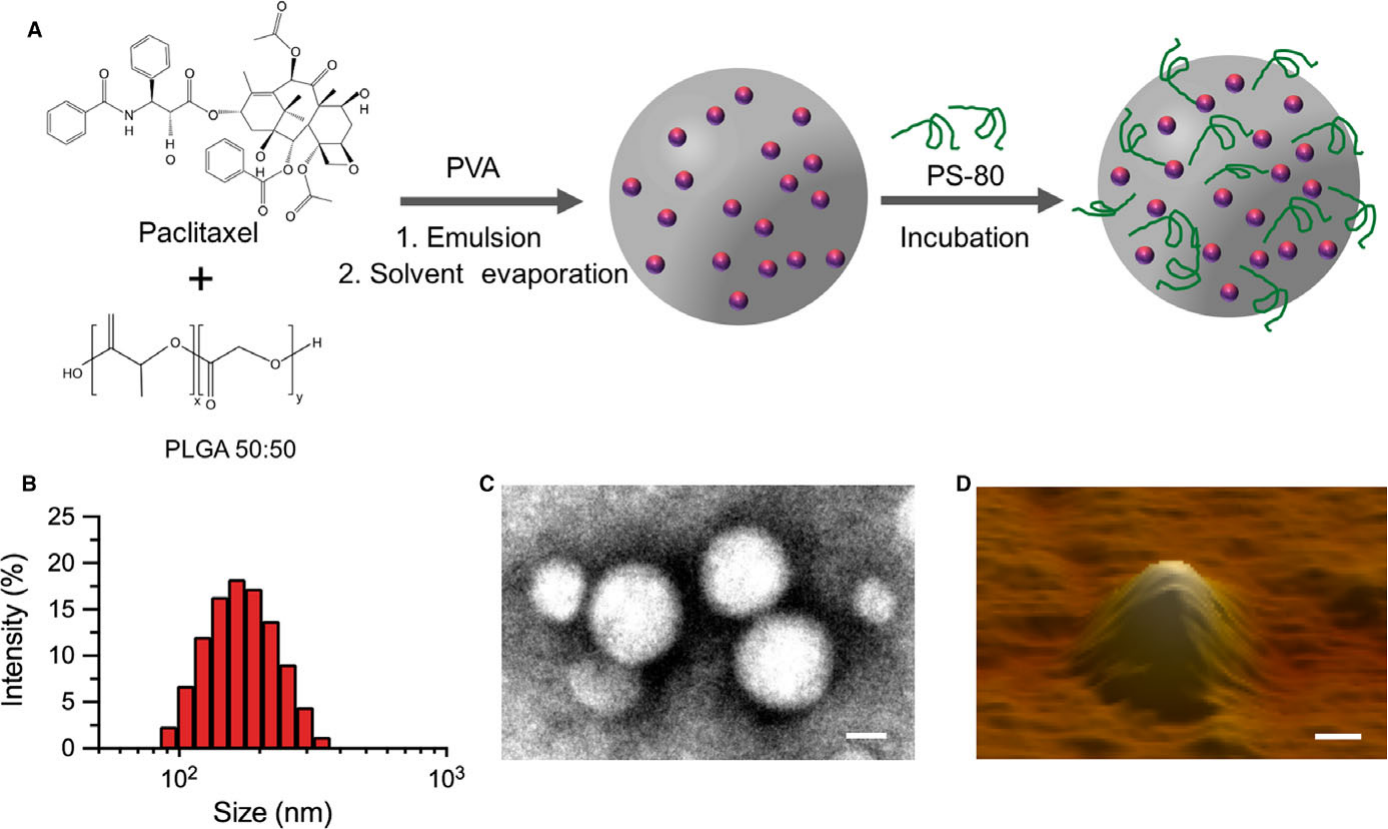
Fabrication and characterization of PPNP. A, Schematic diagram of synthesis of PPNP. B, Histograms of the PPNP size. C, TEM, and D, 3‐dimensional AFM images of PPNP. (C. Scale bar: 100 nm, D. Scale bar: 50 nm)

**Figure 2:**
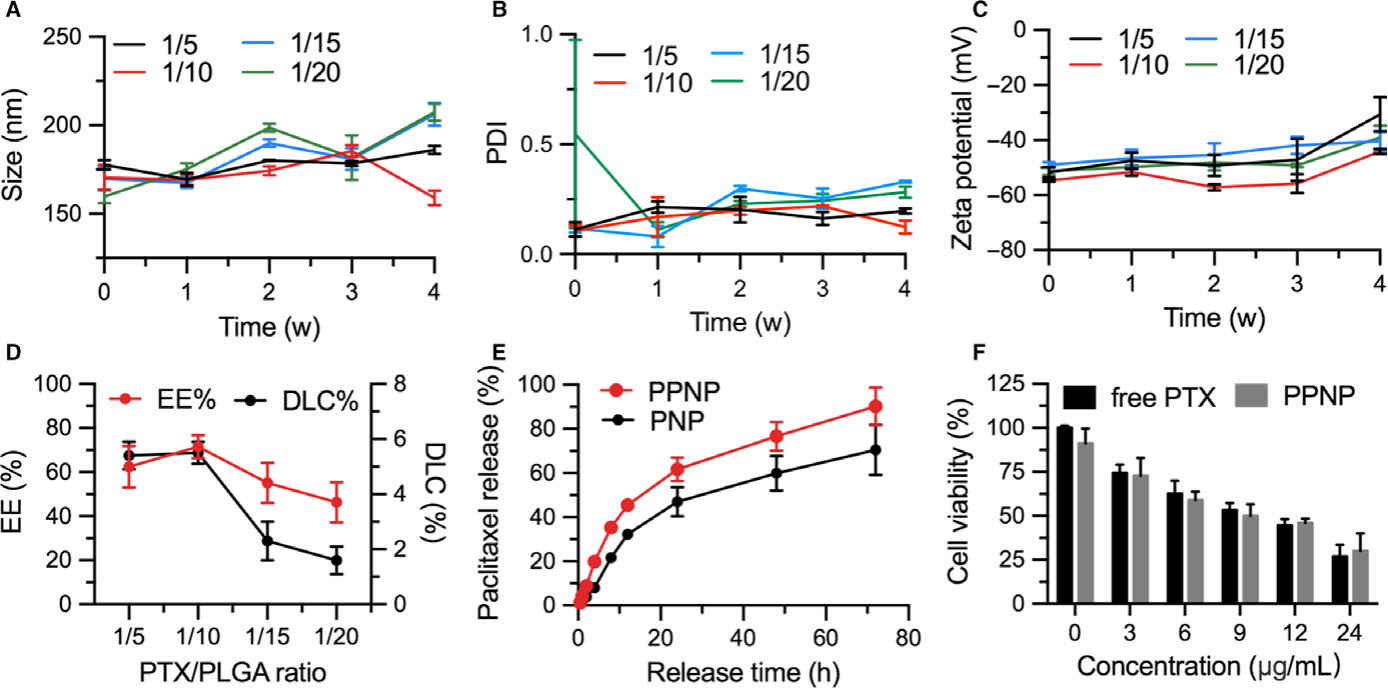
Stability of PPNP over 4 weeks under 4°C. Stability was analysed regarding A, size, B, polydispersity index, and C, zeta potential; n = 3. E, In vitro release of PTX from PPNP. F, Cell viability after treatment with free PTX or PPNP, respectively, at 37°C for 72 h. Data represented mean ± SD (n = 3)

Figure 1A illustrates the schematic diagram to fabricate PPNP through an emulsion/solvent evaporation method, followed by coating with PS‐80. Dynamic light scanning (DLS) measurement showed that the average size diameter of PPNP was 170.5 ± 7.1 nm with a 0.11 ± 0.03 polydiserpersity index (PDI) (Figure 1B). The zeta potential of PPNP was −54.7 ± 0.46 mV. The TEM and AFM images revealed that PPNP appeared a well‐defined spherical morphology and comparative particle size (Figure 1C, D). The stability over time at 4°C were investigated for PPNP, revealing there were not significant changes on the particle size, PDI and zeta potential for 4 weeks (Figure 2A‐C). The drug encapsulation efficiency and loading rate (W/W) varied with the ratio of PTX and PLGA, achieving 62.1% ± 9.5%, 71.6 ± 5.2%, 55.1 ± 9.1% or 46.3 ± 9.2% drug encapsulation efficiency and 5.4 ± 0.5%, 5.5 ± 0.4%, 2.3 ± 0.7% or 1.6 ± 0.5% drug loading rates for the ratio of PTX to PLGA at 1:5, 1:10, 1:15 or 1:20, respectively (Figure 2D). Obviously, higher the PTX encapsulation efficiency and drug loading rate can be achieved when the ratio of PTX to PLGA was at 1:5 or 1:10. Based on the above results, the final ratio of PTX and PLGA at 1:10 was chose for the further studies.

### 3.2 In vitro PTX release and cell viability

Figure 2E demonstrates the release rate of PTX from PPNP was faster than that from PNP which was not coated with PS‐80. About 50% of drug was released from PPNP after 24 hours and nearly 80% after 48 hours, indicating that PS‐80 as a cosolvent/surfactant could favour drug release from NPs. The cell viability revealed PPNP had dose‐dependent cytotoxicity to U87 glioma cells, having a similar toxicity with free PTX at same concentration (*P* > .05; Figure 2F).

### 3.3 Drug delivery across BBB

**Figure 3:**
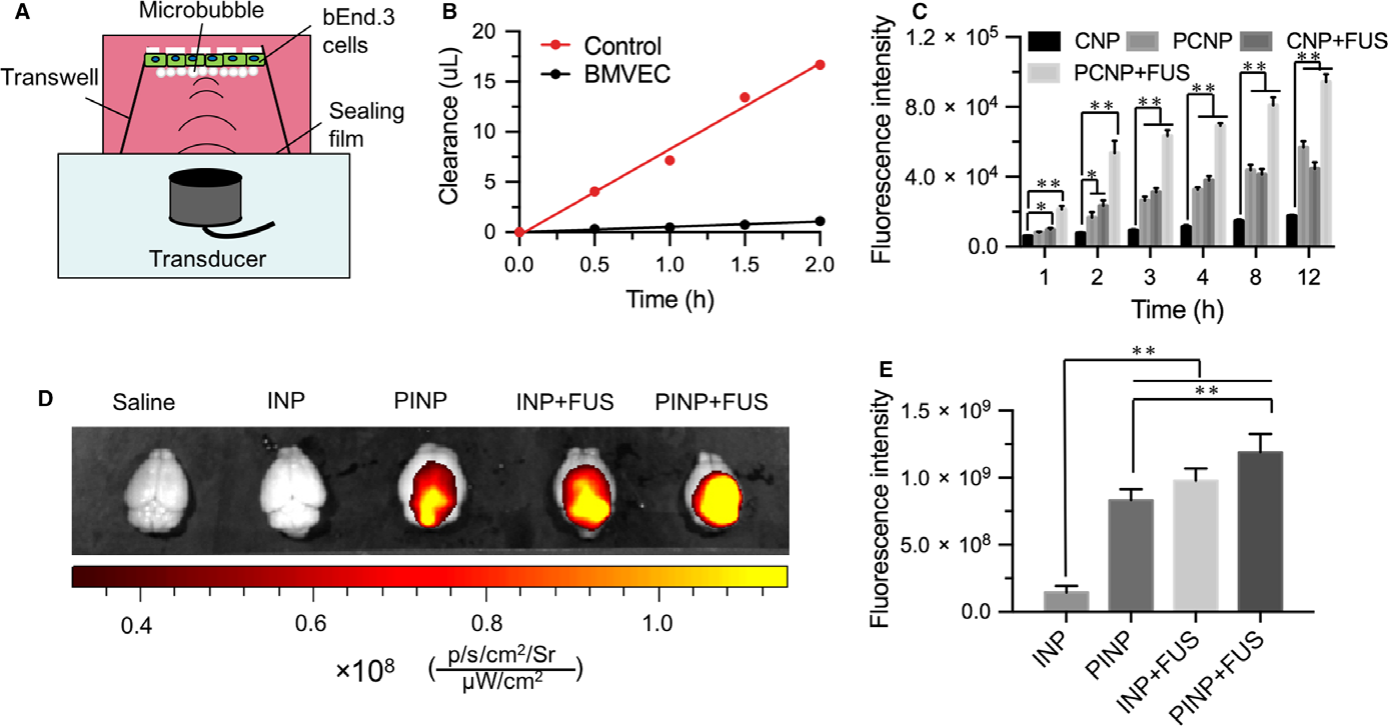
Drug across the in vitro and in vivo blood‐brain barrier (BBB). A, Schematic of FUS‐induced BBB opening in the in vitro model. B, Clearance of fluorescein sodium the in vitro BBB model. C, Quantitative fluorescence intensity in the in vitro BBB model treated with or without CNP and/or FUS (n = 3). D, Fluorescence images of the brains of mice treated with saline, INP, PINP, INP+FUS or PINP + FUS. E, Quantitative analysis of fluorescence intensities from these treated mouse brains. (*P < .05, **P < .001)

To investigate the drug delivery across BBB, we first developed the in vitro BBB model (Figure 3A), in which bEnd.3 cells were seeded onto the plate of transwell. After 9 days, sodium fluorescein barely permeated through the cell barrier, with significantly lower endothelial permeability coefficient (Pflu = (6.87 ± 2.0) × 10^−7^ cm/s) than that of the control (*P* < .001; Figure 3B). The result indicated the in vitro BBB model was successfully developed. After that, CNP and PCNP were examined the in vitro BBB permeability with or without US irradiation. The result showed the nonmodified CNP hardly cross the BBB, just with weak fluorescence on the bottom solution. CNP + FUS or PS‐80 modification to CNP greatly improved BBB permeability, resulting in the stronger fluorescence intensities on the bottom solution (*P* < .01). By contrast, PCNP + FUS produced the highest fluorescence intensities at all examined time (Figure 3C). Quantitatively, the fluorescence intensity of PCNP + FUS was 4.35‐fold, 0.66‐fold, 1.12‐fold higher than these of CNP, PCNP and CNP + FUS at 12 hours, respectively. To confirm the enhanced BBB permeability effect in vivo, the healthy mice were utilized to administrate PINP which contained IR‐780 dye as the model drug and received with FUS, followed by examination with fluorescence imaging. As expected, there did not appear fluorescence signal in the mouse brain when iv injecting INP into mice. Apparent fluorescence could be observed in the brain of mouse received with PINP or INP + FUS, indicating PS‐80 modification or ultrasound treatment can favour nanoparticles cross the BBB. Interestingly, significantly stronger fluorescence signals could be found in the brain of mouse received with PINP + FUS treatment (Figure 3D,E; *P* < .05), revealing the combination of PS‐80 modification with ultrasound treatment can greatly improve the drug BBB permeability efficiency.

### 3.4 Mechanisms of FUS‐assisted BBB opening

**Figure 4:**
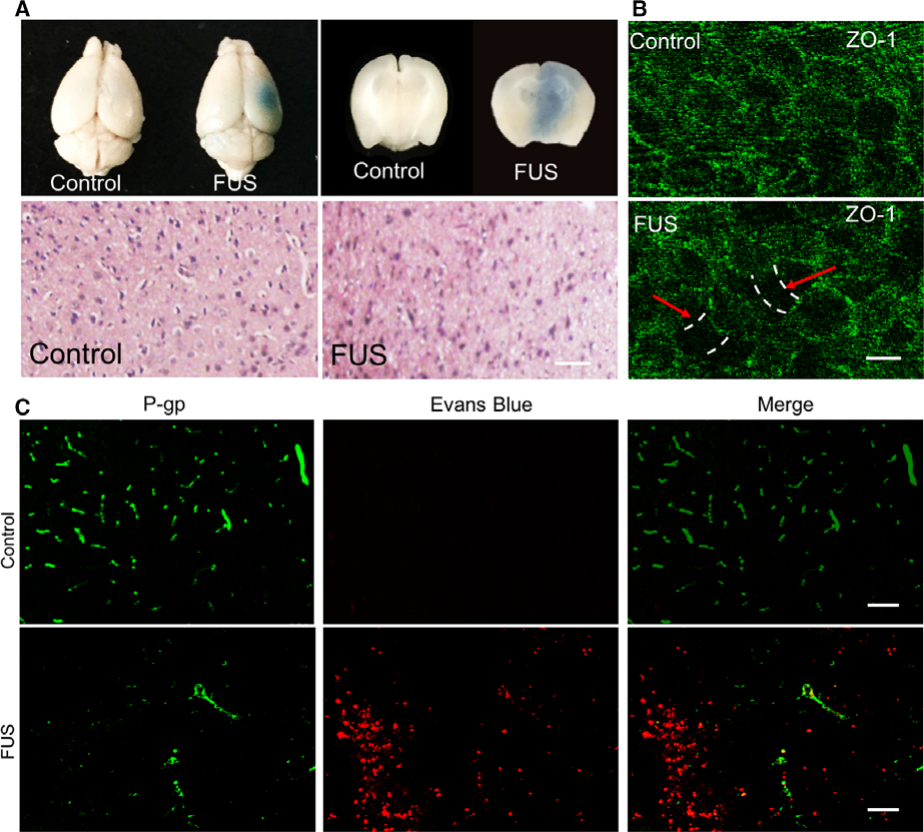
Mechanisms of FUS‐assisted BBB opening. A, Immunofluorescence images of tight junction associated protein ZO‐1 on bEnd.3 cells of control and FUS group. Areas free of cells are marked with an arrow. (Scale bar: 20 μm) B, Evens blue staining of the control and the FUS‐treated brain, confirming FUS‐induced blood–brain barrier opening. (Scale bar: 30 μm) C, Immunofluorescence images of control and FUS groups revealed the reduced P‐gp expression (Green). Red stands for Evans blue. (Scale bar: 50 μm)

Evans blue (EB) staining for the in vivo mouse brain treated with FUS is presented in the Figure 4B, confirming the successful BBB opening (Figure 4A). H&E staining of the FUS‐irradiated brain tissue did not observe the apparent pathological damages such as RBC extravasations, demonstrating the safe and feasible ultrasound parameters used for BBB opening in the study. As tight junction (TJ) is considered as an important structure element regulating the paracellular flux of compounds across the BBB, we tested the integrity of BBB through immunofluorescence staining of ZO‐1 proteins which widely express in brain endothelial cells. The result revealed the disrupted tight junction was obviously visible in the in vitro BBB model when bEnd.3 cells were exposed to FUS irradiation in the presence of MBs (Figure 4B). Given that P‐glycoprotein (P‐gp), overexpressed in the BBB and in most of glioma cells, plays a key role for the drug efflux, we further examined its expression at 24 hours after the FUS treatment. Compared with the control which did not receive FUS irradiation, significantly reduced green fluorescence signals of P‐gp could be observed in the BBB disruption region which could be identified by penetrated Evans Blue emitting red fluorescence (Figure 4C). Thus, our data imply that FUS combined with MBs is really an effective approach to induce the opening of BBB without obvious pathological damages through disrupting tight junction and decreasing the P‐gp expression.

### 3.5 Mechanisms of PS‐80‐meadiated BBB penetration

**Figure 5:**
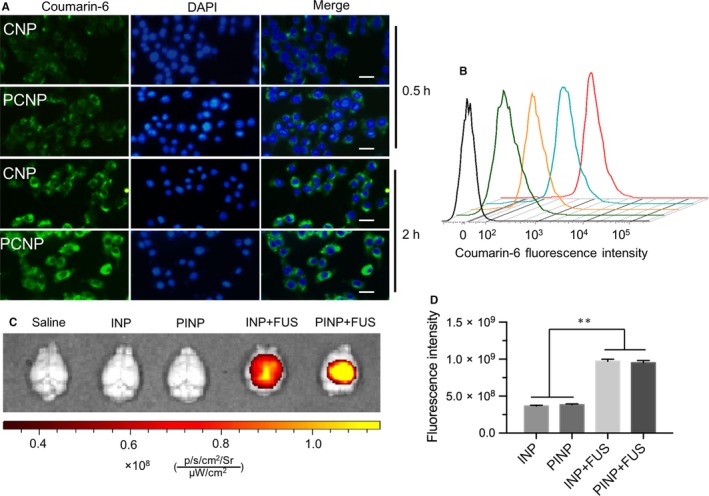
Mechanisms of PS‐80‐meadiated BBB penetration. A, Fluorescence images of bEnd.3 cells incubated with CNP or PCNP after 0.5 and 2 hours. Scale bar: 20 μm. B, The quantitative analysis of cellular fluorescence intensities by flow cytometry. C, Fluorescence images of mouse brains treated with saline, INP, PINP, INP + FUS or PINP + FUS. D, The quantitative fluorescence intensities of brains of ApoE−/− mice after 12 hours with different treatments. **P < .001

To investigate the mechanism of PS‐80‐meadiated BBB penetration, CNP and PCNP encapsulating Coumarin‐6 were used to incubate with bEnd.3 cells, followed by examination with fluorescence microscopy and flow cytometry. The results demonstrated there was significantly stronger Coumarin‐6 fluorescence in the PCNP‐treated bEnd.3 cells than that of CNP‐treated bEnd.3 cells after 0.5‐hour incubation (Figure 5A). The difference of fluorescence intensities became more significant when prolonged exposure time to 2 hours, which were confirmed by flow cytometry (Figure 5B). Notably, there had no significant difference in the fluorescence intensities between the PCNP‐incubated U87 cells and CNP‐incubated cells even after 2 hours treatment (Figure S1). These data indicated that PS‐80 mediates stronger cellular uptake or BBB penetration in a brain vascular endothelial cell‐dependent manner. To prove this speculation, we examined the apolipoprotein E (ApoE) expression between bEnd.3 cells and U87 cells. Data from Western blotting showed significantly higher ApoE expression level in the bEnd.3 cells than that in U87 cells (Figure S2). To further confirm the in vivo mechanism of PS‐80‐mediated BBB penetration, the ApoE knock‐out mice were intravenously administrated with INP or PINP with or without FUS treatment. Similar with the brain of ApoE−/− mice‐treated INP or saline, there were hardly fluorescence signals observable in the brain of ApoE−/− mice received PINP without FUS. But INP or PINP could still penetrate through BBB into the brain of ApoE−/− mice when FUS was utilized (Figure 5C,D), revealing ApoE deficiency did not significantly disturb FUS‐induced BBB penetration for nanoparticles.

### 3.6 In vivo antiglioma efficacy

**Figure 6:**
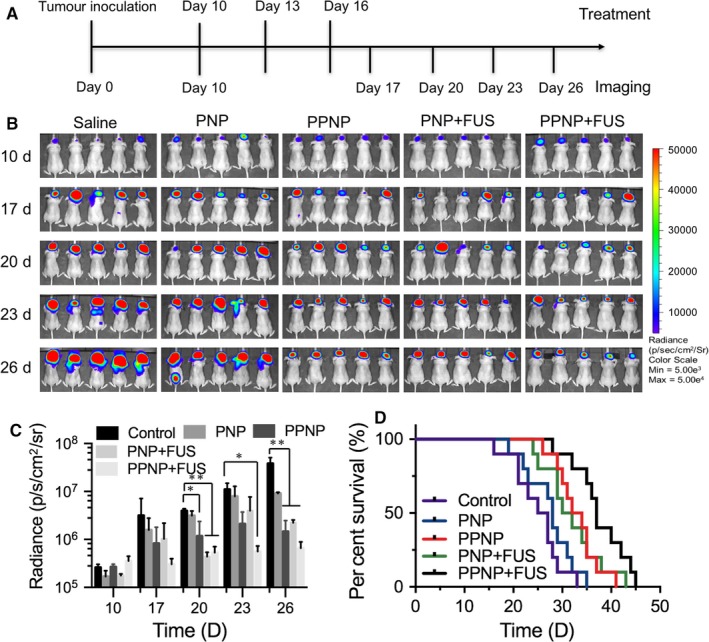
In vivo antiglioma efficacy in the U87‐Luci mouse model. A, Diagram of experimental study over time. B, In vivo bioluminescence imaging for these treated mice and C, quantitative fluorescence intensity of tumour‐bearing mouse on days 10, 17, 20, 23 and 26. D, Kaplan‐Meier survival curves of these treated and control mice. (*P < .05, **P < .001). Data are represented as means ± SD

Next, the in vivo antiglioma efficacy was evaluated using of PNP or PPNP combined with or without FUS. U87‐Luc glioma orthotopic‐transplantation mouse model was constructed and the tumour growth was recorded by an in vivo bioluminescence imaging machine. The experimental scheme over time is shown in Figure 6A. The U87‐Luc glioma‐bearing mice were treated with saline, PNP, PPNP, PNP + FUS or PPNP + FUS, respectively. It can be clearly seen that the group treated with PNP did not exhibit obvious tumour inhibition effect until 26 days when compared to the PBS‐treated group, demonstrating PNP was difficult to deliver into the brain at the early stage of glioma. In comparison with saline‐treated group, the mice treated with PPNP and PNP+US showed significantly stronger tumour growth inhibition effect (*P* < .05). Notably, the group treated with PPNP + FUS showed the strongest tumour growth inhibition effect among all groups (*P* < .01; Figure 6B‐C). Quantitatively, the tumour treated with PNP, PPNP, PNP + FUS or PPNP + FUS treatments was 0.02‐, 0.06‐, 0.04‐, 0.24‐fold lower bioluminescence intensities of saline‐treated tumour by day 26, respectively (Figure 6C). The median survival time of mice treated with PPNP + FUS group increases to 37 days compared with 26 days for control group, indicating systemic administration of PPNP combined with FUS could remarkably improve survival rate and prolong the total survival time.

### 3.7 Histological analysis

**Figure 7:**
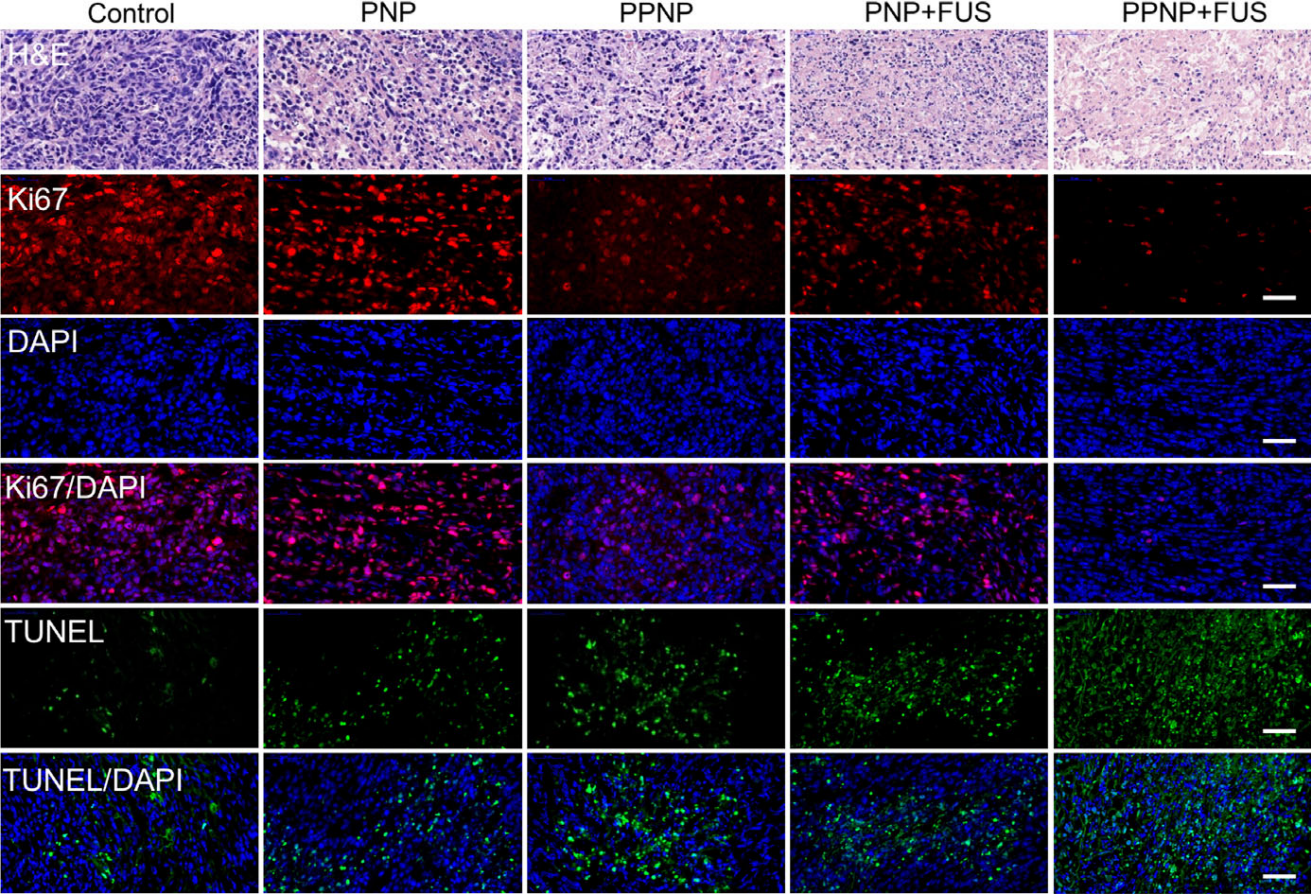
Histological analysis of tumours. H&E staining, Ki‐67 immunofluorescence staining and TUNEL staining of tumours from different treatment groups. Ki‐67‐positive cells were stained red, and the apoptotic cells were stained green. Cell nuclei were stained by DAPI for blue. Scale bar: 50 μm

Histological analysis was performed to evaluate the antitumour efficacy. Haematoxylin and eosin (H&E) staining revealed the tumour cells from PPNP + FUS‐treated mouse brain significantly decreased and the tissues trended to be normalized, compared with other 4 groups. Immunofluorescence staining of Ki‐67, a marker protein to show the tumour cell proliferation potential, demonstrated there were the lowest levels of Ki‐67‐positive cells in the tumour exposed to PPNP + FUS. On the contrary, the PPNP+FUS‐treated mouse tumour showed a large number of TUNEL‐positive cells, indicating the presence of more apoptotic tumour cells within the tumours (Figure 7). Pathological analysis of the main organs from these treated mice did not find any appreciable abnormality (Figure S3). Obviously, PPNP combined with FUS‐induced BBB opening was tolerable and had no obvious acute toxicity to the mice.

## 4 DISCUSSION

Failed or limited drug penetration through BBB brings with greatly challenge for glioma treatment. Great efforts have been attempted to modify the drug formulations to overcome the barrier but limited progress has made. Recently, PS‐80 brings with new tool to address this issue through activating ApoE‐mediated transport pathway.^21^, ^22^, ^23^ However, PS‐80‐meadiated whole brain drug delivery unavoidably brings some risks for glioma patients and was invalid for these glioma patients with low ApoE expression or ApoE functional defects. In this study, we fabricated the PS‐80‐modified PTX‐loaded PLGA nanoparticles and combined it with FUS to enhance the local drug delivery into the brain tumour. To ensure the full contact between PPNP and vascular endothelial cells, in this study we chose to use the separate PPNP and MBs, but not conjugate these PPNP on the surface of MBs. Obviously, there are several advantages in our brain drug delivery platform over the previous reports. The one is to improve the local tumour delivery efficiency, making it possible to reduce the drug dose and to decrease the side effect. The other one can benefit more glioma patients who have low ApoE expression or ApoE functional defects.

In this study, we select PTX as the model drug because PTX has been demonstrated to be high tumour‐killing activity against various kinds of tumours in both preclinical and clinical studies.^38^, ^39^, ^40^, ^41^, ^42^, ^43^ Great potentials for PTX have been proven in the glioblastoma multiform (GBM) cells by inducing mitotic slippage, only just requiring a low drug concentration to kill tumour cells.^44^ Unfortunately, PTX is difficult to cross the BBB, greatly reducing its efficacy against orthotopic glioma in vivo. In the current study, we encapsulated the PTX into PLGA nanoparticles and further coated these particles with PS‐80 surfactant, thus obtaining PPNP (Figure 1A). The resulting PPNP not only had good stability and fast drug release rate, but also had significant toxicity to U87 cells (Figure 2).

FUS‐induced BBB opening has many unique advantages such as noninvasiveness, temporary and local BBB opening, providing a promising method for enhancing the targeted delivery of therapeutic agents into tumour site. In this study, our results showed that FUS in the presence of MBs can effectively induce BBB opening and locally deliver nanoparticles in vitro and in vivo models (Figure 3). Immunofluorescence staining of ZO‐1 and P‐gp revealed FUS can temporarily disrupt TJs of BBB and reduce P‐gp expression (Figure 4), demonstrating both mechanical and biological effects are involved in the FUS‐induced BBB opening. On the one hand, the physical mechanical effects produce the micro‐scale pores or increase the gap between endothelial cells because of the bubble cavitation. On the other hand, such mechanical force also reduces the level of P‐gp proteins, decreasing the drug efflux from the cells, in agreement with the previous report.^36^ Moreover, our data also showed that the PS‐80 modification of largely favoured these nanoparticles to permeate through the BBB in a ApoE‐dependent manner (Figure 5), indicating that ApoE protein plays an important role in PS‐80 mediated BBB penetration. Nicoll demonstrated that ApoE is involved in the delivery of lipids to tumour cells and in the recycling of lipids by macrophages, raising the possibility that ApoE‐mediated transport in brain tumours.^45^ Mechanisms study demonstrated ApoE proteins possess extraordinary features which is readily assembled with hydrophobic compounds *via* its compact hydrophobic units. These assemblies can then be converted to stable particles by protein‐protein interactions *via* coiled coil regions which exist in ApoE structure.^46^

To improve the drug local delivery and enhance chemotherapeutic efficacy against brain tumour, in this study, we utilized FUS‐combined MB cavitation to induce the BBB opening, giving the PS‐80 modified nanoparticles higher efficiency to cross the BBB. Data from the in vivo antitumour experiment demonstrated that PPNP + FUS group has the stronger antitumour efficacy when compared to the FUS + PNP or only PPNP groups (Figure 6). Longer survival time was also observed in tumour‐bearing mice treated with PPNP + FUS, suggesting that FUS combined with PS‐80 modification is significantly more efficient to deliver drugs into the brain tumours than either of them. Indeed, the enhanced antitumour effect was confirmed by histological analysis, revealing an increased apoptosis and decreased proliferation in tumour xenografts treated with PPNP + FUS (Figure 7). Thus, our study provides a new strategy to efficiently and locally deliver drugs into the brain to treat glioma through combining FUS with PS‐80‐modification of nanoparticles. As schematically shown in Figure S4, FUS can activate the MBs to produce cavitation, resulting in the disruption of TJs of BBB and enlargement of the endothelial cell gaps, by which PPNPs can enter into the brain. Also, the down‐regulated expression of P‐gp from FUS cavitation would cause reduced drug efflux. In addition, PPNP can attach with ApoE receptor and then activate receptor‐mediated endocytosis. All of the factors collectively contribute to the enhanced BBB permeation and antitumour efficacy.

## 5 CONCLUSIONS

In this study, we successfully fabricated PPNP and locally deliver these nano‐drugs into the brain tumour. Also, we demonstrated the enhanced drug delivery mechanisms which can contribute to the temporary disruption TJs of BBB and reduction in P‐gp expression from FUS‐induced BBB opening and the PS‐80‐meadiated ApoE‐dependent permeation. In conclusion, our study provided a new strategy to efficiently and locally deliver drugs into the brain to treat glioma.

## CONFLICTS OF INTEREST

The authors declare no conflicts of interest.

## AUTHOR CONTRIBUTIONS

Y.L. and M.W. contributed equally. F.Y. and H.Z. conceived and designed the experiments. Y.L., M.W., N.Z., C.T. and X.L. performed the experiments. F.Y. and M.W. analysed the data. F.Y. wrote the manuscript. All authors discussed the results and commented on the manuscript.
