# Supplementary material for: Mechanisms of enhanced antiglioma efficacy of polysorbate 80‐modified paclitaxel‐loaded PLGA nanoparticles by focused ultrasound
Source: J Cell Mol Med. 2018 Jun 29;22(9):4171–82. doi: 10.1111/jcmm.13695 (PMC6111803; doi:10.1111/jcmm.13695)
Supplement: Supplementary file 1 [file JCMM-22-4171-s001.doc]

**Supplementary material**

**Mechanisms of Enhanced Anti-glioma Efficacy of Polysorbate 80-modified Paclitaxel-loaded PLGA Nanoparticles by Focused Ultrasound**

Yingjia Li ^a, 1^, Manxiang Wu ^a, 1^, Nisi Zhang ^b,c^, Caiyun Tang ^b,d^, Peng Jiang ^e^, Xin Liu ^b^, Fei Yan ^b,*^, Hairong Zheng ^b^

*1. Department of Medicine Ultrasonics, Nanfang Hospital, Southern Medical University, Guangzhou, China;*

*2. Paul C. Lauterbur Research Center for Biomedical Imaging, Institute of biomedical and Health Engineering, Shenzhen Institutes of Advanced Technology, Chinese Academy of Sciences, Shenzhen, China;*

*3. Biomedical Engineering Department, College of Engineering, Peking University, Beijing, China;*

*4. Pharmaceutical Analysis Department, College of Pharmacy, Jiamusi University, Jiamusi, China;*

*5. Shenzhen Key Laboratory of Nanobiomechanics, Shenzhen Institutes of Advanced Technology, Chinese Academy of Sciences, Shenzhen, China*

^1^ These authors contributed equally to this work.

*Corresponding authors at: Shenzhen Institutes of Advanced Technology, Chinese Academy of Sciences, Shenzhen, 518055, China.

E-mail address: [fei.yan@siat.ac.cn](mailto:hr.zheng@siat.ac.cn) (F. Yan). Tel: +86 755 86392284 Fax: +86 755 96382299


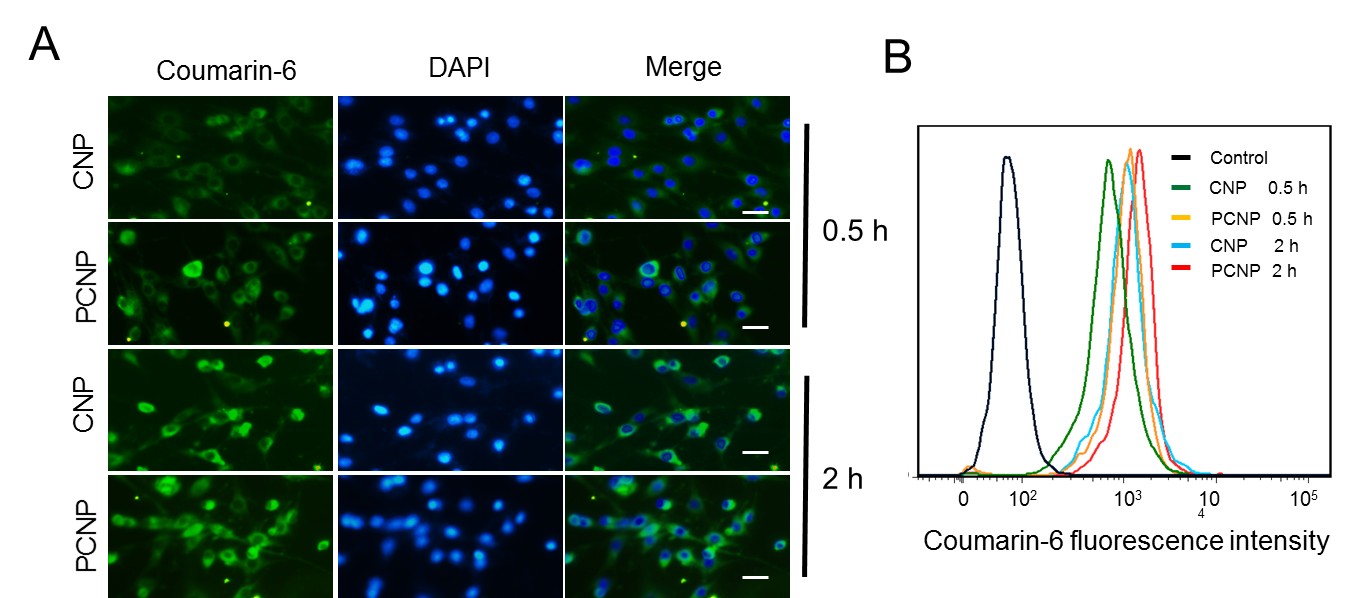


Figure S1. The uptake analysis of U87 cells. (A) Fluorescence microscopy images of intracellular Coumarin-6 distribution in U87 cells after 0.5 h and 2 h incubation with CNP or PCNP. Scale bar: 20 µm. (B) Flow cytometry of these cells after 0.5 h and 2 h incubation.


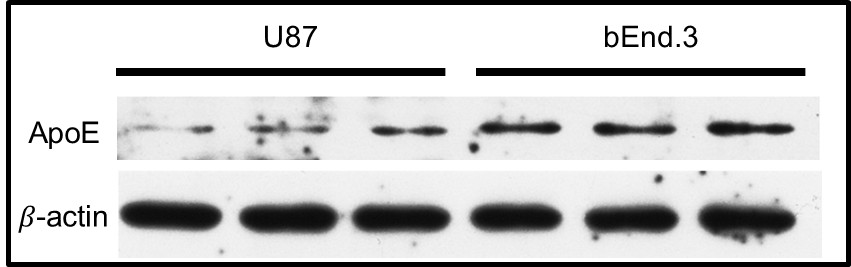


Figure S2. Western blot analysis of U87 cells and bEnd.3 cells. Panel 1-3 for the U87 cells and Panel 4-6 for bEnd.3 cells for triple repetition.


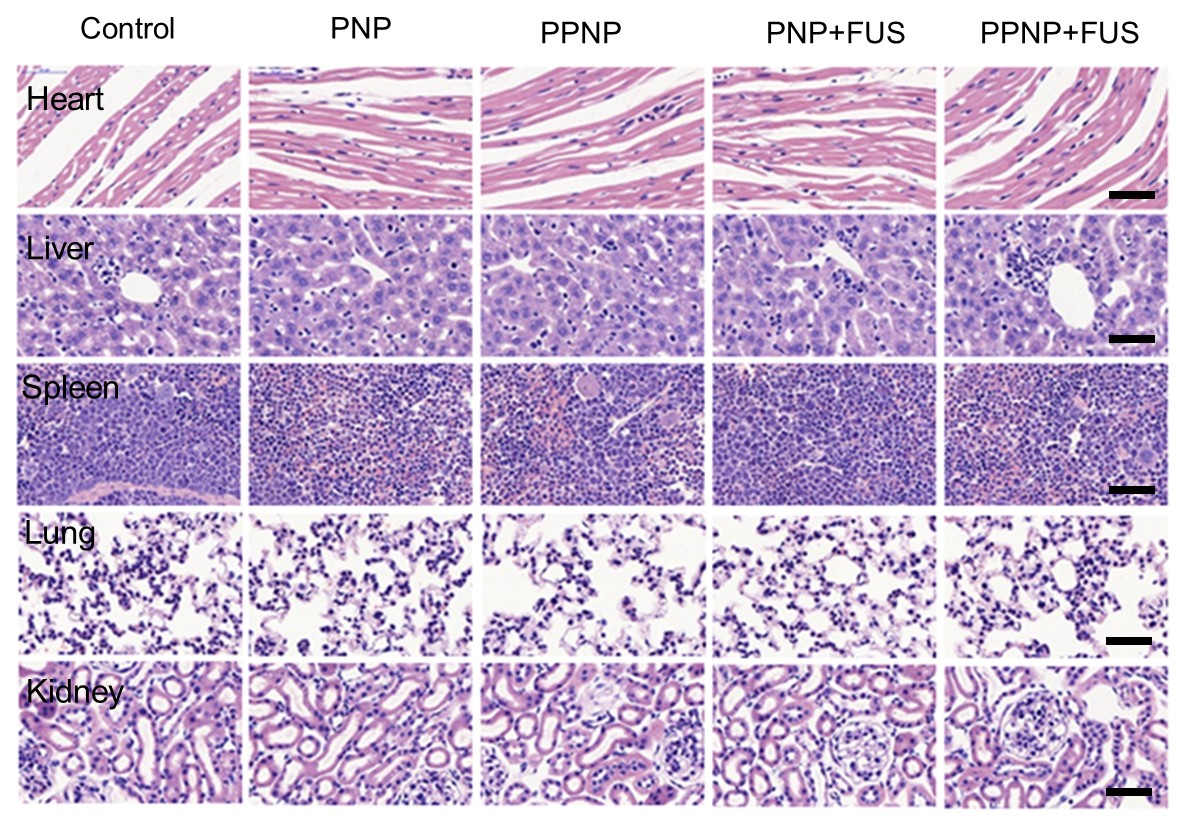


Figure S3. H&E stained images of major organs from mice treated with PBS, PNP, PPNP, PNP+ FUS or PPNP+FUS, respectively. Scale bar, 50 μm.


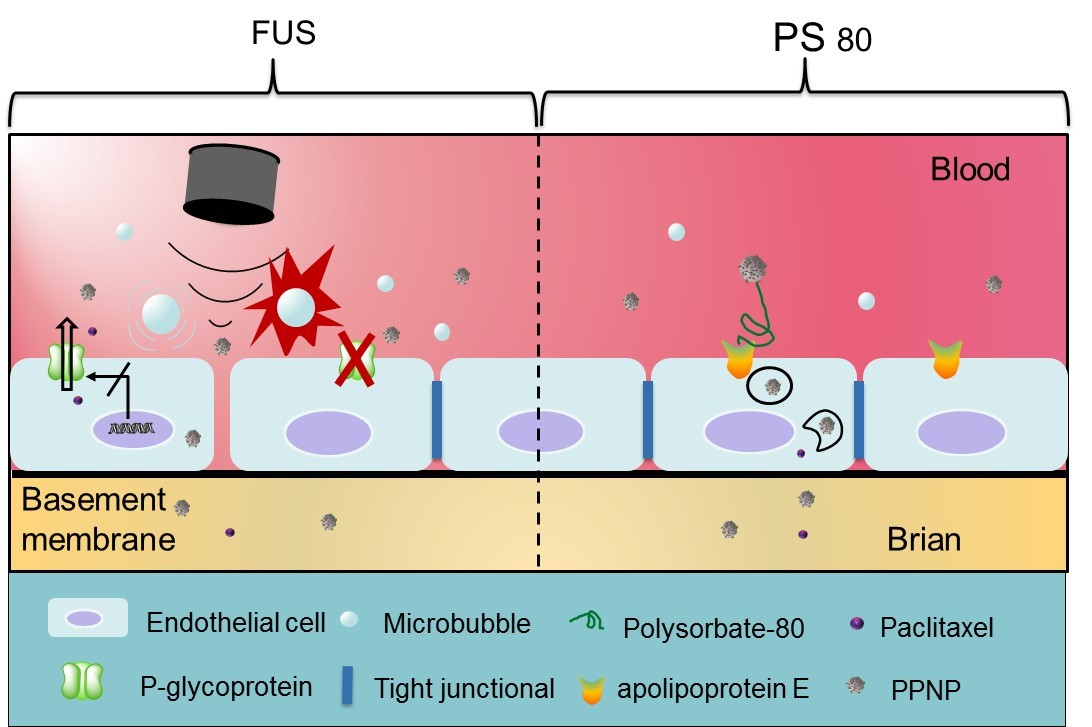


Figure S4. Schematic illustration of mechanisms for FUS-induced BBB opening and enhanced PPNP delivery across BBB into brain tumor.
